# Description of a new natural *Sonneratia* hybrid from Hainan Island, China

**DOI:** 10.3897/phytokeys.154.53223

**Published:** 2020-08-03

**Authors:** Cairong Zhong, Donglin Li, Ying Zhang

**Affiliations:** 1 Life Science and Technology School, Lingnan Normal University, Zhanjiang, Guangdong 524048, China Hainan Academy of Forestry, Hainan Mangrove Research Institute Haikou China; 2 Hainan Academy of Forestry, Hainan Mangrove Research Institute. Haikou, Hainan, 571100, China Shaoguan University Shaoguan China; 3 College of Ying-Tong Agricultural Science and Engineering, Shaoguan University, Shaoguan, Guangdong 512005, China Lingnan Normal University Zhanjiang China

**Keywords:** *
Sonneratia
*, new hybrid, Dongzhai Harbour, Hainan Island

## Abstract

Here, we describe, illustrate and compare a new natural hybrid, Sonneratia × zhongcairongii Y. S. Wang & S. H. Shi (Sonneratiaceae), with its possible parent species. Based on its morphological characteristics and habitat conditions, this taxon is considered to represent a sterile hybrid between *S. alba* and *S. apetala.* In China, the new hybrid is only reported in the mangrove forest in Dongzhai Harbour, Hainan Island. It has intermediate characteristics with its parents by elliptical leaf blades, peltate stigma, terminal or axillary inflorescence with 1–3 flower dichasia, cup – shaped calyx (4–6 calyx lobes) and no petals. We also provide a key for the identification of *Sonneratia* species.

## Introduction

Sonneratiaceae is a small tropical plant family consisting of only two small genera, *Sonneratia* and *Duabanga*. The inland genus *Duabanga* is an evergreen component of the rainforest belt, comprising two species within a more restricted range in Malaysian, Indonesia and China (Tomlinson 1986; Goutham-Bharathi et al. 2012). *Sonneratia*, a genus of mangroves, is one of the most important components of the intertidal zones of the tropical and subtropical coastal regions, ranging from East Africa through Indo-Malaya to tropical Australia and into Micronesia and Melanesia (Tomlinson 1986). This genus is also well-adapted to harsh intertidal zones with high salinity, hypoxia and ultraviolet (UV) radiation (Duke et al. 1998).

*Sonneratia* consists of six species and three interspecific hybrids (Duke and Jackes 1987; Duke 1994; Goutham-Bharathi et al. 2012; Yang et al. 2016). Amongst these, *S. alba*, *S. caseolaris*, *S. ovata* and S. × gulngai are the most widespread species (Tomlinson 1986; Goutham-Bharathi et al. 2012; Yang et al. 2016), whereas *S. lanceolata* and S. x urama are strictly limited to north-western Australia, southern New Guinea and a few locations in Indonesia (Yang et al. 2016). *S. griffithii* has a restricted distribution along the shores of the Andaman Sea, north to Bengal and south to the upper Malay Peninsula (Tomlinson 1986). S. x hainanensis, a hybrid derived from the cross between *S. alba* and *S. ovata*, is found in Hainan, China (Ko 1985; Wang et al. 1999). It was first reported that S. x hainanensis was in north-western Borneo as a nom. nud., based on morphological and cytological analyses (Muller and Hou-Liu 1966; Zhou et al. 2005). The parents of S. x hainanensis are widely distributed; however, more collections are needed. The mangrove *S. apetala* is restricted to southern India and Burma and is the most distinctive species because of its mushroom-like stigma (Tomlinson 1986). In China, *S. apetala* was first introduced in Dongzai Harbour, Hainan Island in 1985 from Bangladesh (Peng et al. 2012). Due to its accelerated growth and high tolerance of environmental stresses, *S. apetala* has been used as the pioneer species for mangrove restoration in estuarine and coastal areas. The species, *S. alba*, is an endemic species on Hainan Island (Li et al. 2017). Between two mixed populations, plants with intermediate characteristics have recently been encountered that obviously belong to the hybrid. In this study, we describe the new hybrid, S. x zhongcairongii and its features that distinguish it from both parent species.

## Materials and method

The morphology of *Sonneratia* species presented here is based on field, vegetative and reproductive characteristics. Field traits were recorded on site, whereas vegetative and reproductive characteristics were observed and measured using fresh specimens, material preserved in 70% ethanol or press-dried specimens. Digital calipers (Mitutoyo, Japan) and a dissecting microscope with calibrated eye (Olympus, Germany) were used to describe the detailed morphological characteristics of samples. All photographs were taken in the field, i.e. in the natural habitat of the species, using a digital camera (cannon EOS RP, Japan). The morphological characteristics of *Sonneratia* species in Hainan Island have been summarized in a key to facilitate identification.

## Results

**Figure 1.**
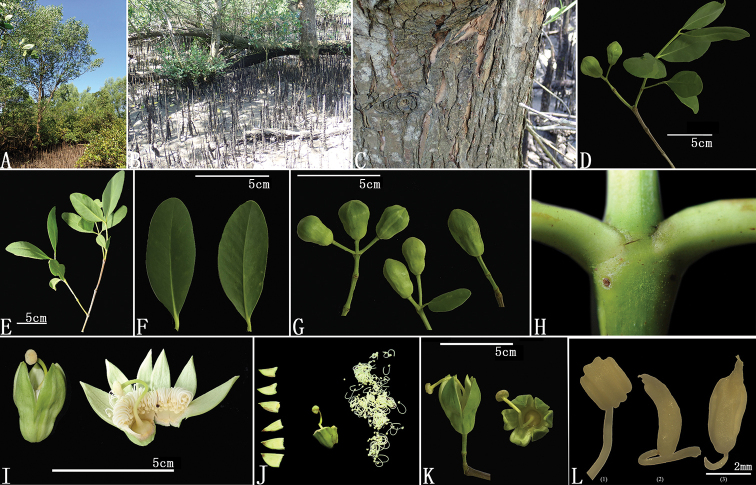
Morphology of S. × zhongcairongii. **A** Habitat **B** stem with aerial root **C** bark **D** branches **E** leaf branch end with flowers **F** leaves **G** inflorescence **H** minute bract at a dichotomous inflorescence branch **I** flower **J–L**. Dissection of the flower (**J**), pistil (**K**) and stamens (**L**).

**Figure 2.**
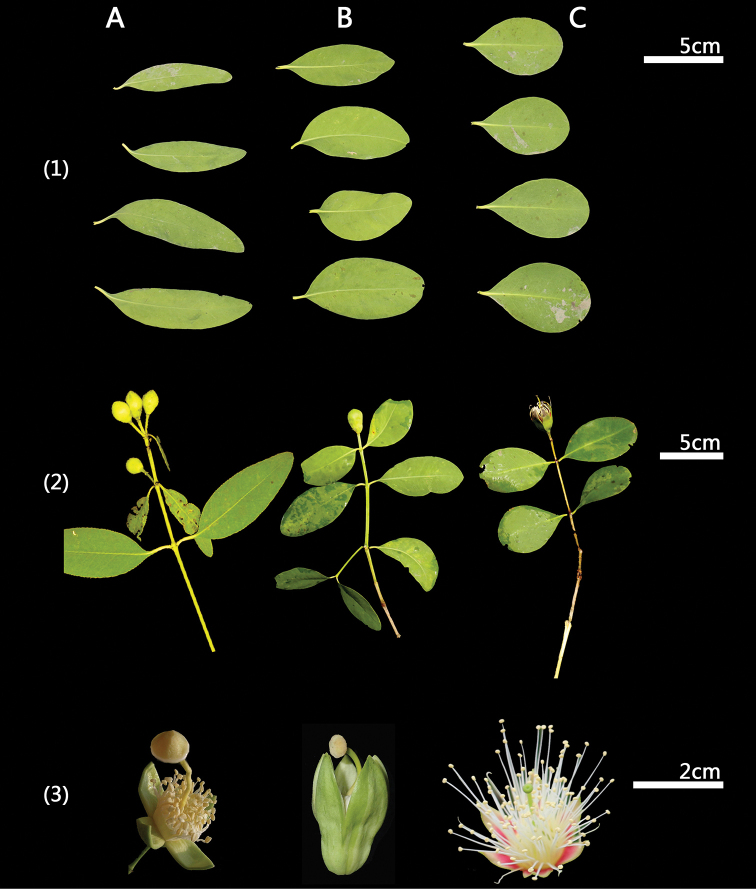
Comparison of the three taxa A *S. apetala* B S. × zhongcairongii C *S. alba***1** leaves, **2** branches, **3** flowers.

**Figure 3.**
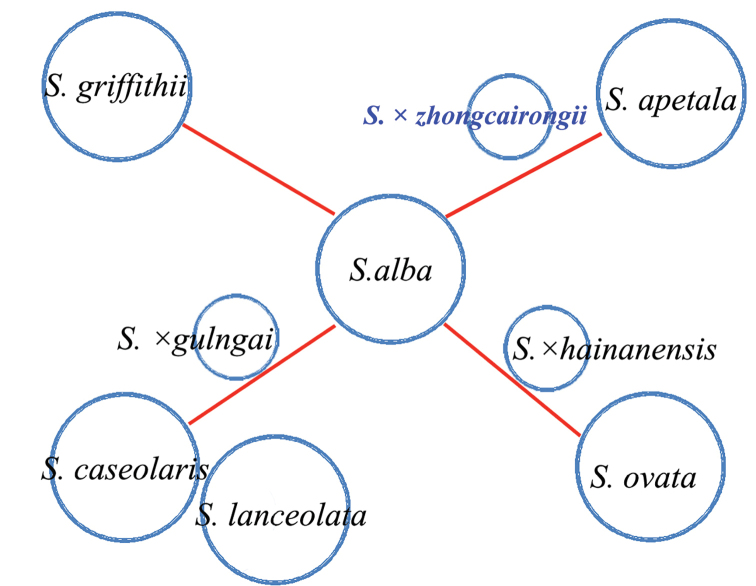
Schematic diagrams of *Sonneratia* taxa and their inter-specific affinities deduced from morphometric analyses. The choice of circle size and line length is arbitrary.

The results of morphometric analysis showed that S. x zhongcairongii is more similar to its parents *S. alba* and *S. apetala* than to other *Sonneratia* taxa (Fig. 1, Table 1). Additionally, the morphology analysis of S. x zhongcairongii was intermediate between that of its parents (Figs 2, 3). The flowers of S. x zhongcairongii contained several abnormally-developed anthers (Fig. 1L), which might account for the 100% abortion rate and consequently the lack of fruit and seed (Table 1).

**Table 1.** Comparison of described *Sonneratia* taxa and the new Hainan material.

Character	S. × zhongcairongii	*S. alba* ^[1-2]^	*S. apetala* ^[3]^	*S. caseolaris* ^[1-2]^	*S. × gulngai* ^[1-2]^	*S. lanceolata* ^[1-2]^	*S. ovata* ^[1,3]^	*S. × hainanensis* ^[4]^	*S. griffithii* ^[3,5]^
Leaf blades	elliptic	obovate or elliptic to ovate	narrowly elliptic to lanceolate	elliptic	elliptic	elliptic	broadly ovate	elliptic or broadly elliptic	obovate or suborbicular
Leaf apices	rounded mucronate	rounded	rounded mucronate	apiculate, mucronate	apiculate, mucronate	apiculate, mucronate	rounded	rounded	obovate mucronate
Leaf base	attenuate oblique	attenuate oblique	attenuate oblique	attenuate oblique	attenuate oblique	attenuate oblique	reniform	broadly cuneate	cuneate
Peduncle	terete	terete	terete	terete or tetragonous	terete	terete or tetragonous	terete	terete	terete
Calyx lobes	4~6; inner often green	6~7(8); inner often red	4; inner often green	5~7; inner often red-streaked	5~7; inner often green	5~7; inner rarely red-streaked	6; inner often red at base	6; inner often red	6-7: inner often green
Petals	absent	white^[6]^, linear-spathulate	absent	red, linear	red, linear	red, linear, rarely double	absent	white	white (absent)*
Stamen	white	white	white	red, rarely white	red	white	white	white	white
Stigma	Mushroom-like, to 5~7 mm wide	capitate but not expanded, 1~3 mm wide	Mushroom-like, to 7~10 mm wide	capitate but not expanded, to 3 mm wide	capitate but not expanded, to 1.7 mm wide	capitate but not expanded, to 3 mm wide	capitate but not expanded, to 3 mm wide	capitate but not expanded, to 3 mm wide	capitate but not expanded, to 3 mm wide
Inflorescence	terminal or axillary, 1-3(-5)-flowered dichasia	terminal cyme occur either solitarily or in groups of three	terminal cyme from branch axis	terminal or axillary, 1-3(-5)-flowered dichasia	terminal or axillary, 1-3-flowered dichasia	terminal or axillary,1(-2)-flowered dichasia	terminal cyme or solitarily or in groups of three	terminal cyme 1-3(-5)-flowered dichasia	terminal cyme 1(-2)-flowered dichasia
calyx (hypanthium)	cup-shaped	cup-shaped	flat-expanded	flat-expanded	cup-shaped	flat-expanded	cup-expanded	cup-shaped	cup-shaped
Fruit	Not developed	Width = corolla width	Width = corolla width	width 5 mm > corolla width	Width = corolla width	width 5 mm > corolla width	width 6-8 mm > corolla width	width 5 mm > corolla width	Width = corolla width
Seeds	Not developed	falcate	falcate	angular irregular	angular irregular	angular irregular	rounded irregular	angular irregular	angular

Taken from ^[1]^Duke and Jackes (1987), ^[2]^Duke (2006), ^[3]^Goutham-Bharathi et al. (2012), ^[4]^Ko (1993), ^[5]^Tomlinson (1986), ^[6]^Wang and Wang (2007).

### Taxonomic treatment

#### S. × zhongcairongii

Taxon classificationPlantaeAmmonoideaHoplitidae

Y. S. Wang & S. H. Shi nothosp. nov.

9CE00772-8AAA-5387-BF07-A87DD0D9C2B3

Figure 1

##### Material.

Dongzhai Harbour, mangrove forest Hainan Island, China (Fig. 1A), 19°58'12"N, 110°34'48"E, 13 June 2018, Cairong Zhong, No. Saa20180613-001 (Holotype: IBSC; Isotype: IBSC).

##### Morphological traits.

Trees, evergreen, 10–12 m tall, highly branched (Fig. 2A). Bark smooth or lightly fissured flaky, dark grey to pale fleshy green; stem base simple. Leaves simple, opposite, leaf blade leathery, glabrous, pale green, elliptical, 2–9 cm long,1–5 cm wide, apex obtuse, base acuminate, margin entire; petiole 0.3–1 cm; stipules absent. Inflorescence terminal or axillary, 1–3 or 1–5 flowered dichasia; flower bud ellipsoidal, 1.5–2.4 cm in length, 1–1.5 cm width, constricted medially, green, glossy, smooth, slightly angular; Calyx cup-shaped, lobes 4–6, wide ovate (0.8–1.2 cm long, 0.5–0.8 cm wide), apex acute, inner often fleshy green inside. Petals absent; stamens numerous along calyx, white, 1–1.5 cm in length; stigma peltate to 5 mm wide. Fruits not developed.

##### Distribution.

The hybrid is currently found only in Dongzhai Harbour within an area of 48 m^2^, mangrove forest, Hainan Island, China.

##### Habitat and ecology.

The hybrid grows in a mangrove forest.

##### Phenology.

The new hybrid flowered from the beginning of March to the end of October.

##### Conservation status.

The new hybrid S. × zhongcairongii was collected only from the mangrove forest in Dongzhai Harbour. At this site, only two individuals were observed.

## Discussion

To date, only three hybrids including S. × zhongcairongii have been reported in the genus *Sonneratia*. As with S. × zhongcairongii, other two hybrids have restricted location in the cross distribution of each parents (Duke and Jackes 1987; Duke 1994; Goutham-Bharathi et al. 2012; Yang et al. 2016). Only two individuals of the new hybrid were observed in China. The parent, *S. apetala*, is an exotic species in China, whose mixed location with *S. alba* started from 1985 (Peng et al. 2012). The morphological characteristics of S. × zhongcairongii were intermediate between its parents (Figs 2, 3); this result is consistent with the other two *Sonneratia* hybrids (Tomlinson 1986; Duke and Jackes 1987). S. × zhongcairongii showed complete abortion. However, on the other two hybrids (*S. × gulngai* and S. × hainanensis) can be found fruit and seeds with heavy abortion degrees (Tomlinson 1986; Wang and Wang 2007).

Backer and van Steenis (1951) compiled a thorough review of the Sonneratiaceae, a family of the order Myrtales. Two genera were described and include *Duabanga* and *sonneratia*. Gao Yunzhang divided the Sonneratia genus into two sections, sect. Sonneratia and sect. Pseudosonneratia, based on the presence or absence of petals (Ko 1985) and which was also used in the research of *sonneratia* Linn. in Australia, New Guinea and the south-western Pacific region (Backer and van Steenis 1951). By adding one new species found in China (*S. paracaseolaris* Ko, E. Y. Chen et W. Y. Chen), Gao Yunzhang regrouped the *Sonneratia* species in China (Ko 1993). Subsequently, a detailed anatomical analysis containing morphology of leaf, flower, fruit, seed and wood of five species of *Sonneratia* Linn. in China showed that the use of petal presence or absence was appropriate to distinguish species in *Sonneratia* Linn. (Chen 1996). Duke and Jackes worried about the use of petal presence or absence to distinguish between apetalous *S. alba* with *S. ovata* which was found to be less common, normally apetalous (Duke and Jackes 1987). Then the wrong character of *S. alba* was revised from apetalous to white, linear-spathulate (Wang and Wang 2007). Compared with characters of petal, stamen, leaf and flower bud, the stigma morphological characteristics have been used to group nine species and hybrids in *Sonneratia* Linn. (Wang and Chen 2002). In this study, we combined the use of petal presence or absence and stigma morphological characteristics to regroup *Sonneratia* plants and the new hybrid was most closely related to one of its parents, *S. apetala*.

To better distinguish amongst species belonging to the genus *Sonneratia*, we created a classification as shown in Table 1. The distribution range of the hybrid S. × zhongcairongii often overlaps with that of *S. alba* Smith. and *S. apetala* Buch. -Ham., which provides the possibility of formation of the hybrid species. The same is true for S. × gulngai N. C. Duke, S. × hainanensis Ko, E. Y. Chen et W. Y. Chen (Wang et al. 1999). The overlapping distributions of parent species contributed to the greater opportunity to form a natural hybrid (Zhou et al. 2008). Interestingly, one of the parents of all three hybrids is *S. alba*, which may be due to the fact that *S. alba* is a widely-distributed species, although further investigation is needed to determine the exact reason.

### Key for the classification of *Sonneratia* species in China

**Table:** 

1	Petals present	**2**
–	Petals absent	**3**
2	Petals white	**4**
–	Petals red	**5**
3	Stigma capitate but not expanded	**6**
–	Stigma mushroom-like	**7**
4	Leaf blades obovate or elliptical to ovate	***S. alba***
–	Leaf blades elliptic or broadly elliptical	***S. × hainanensis***
5	Fruit calyx flat-expanded, fruit width > corolla width by 5 mm	**8**
–	Fruit calyx cup-shaped, Width = corolla width	***S. × gulngai***
6	Leaf blade apices rounded	***S. ovata***
–	Leaf blade apices obovate mucronate	***S. griffithii***
7	Flat-expanded calyx, fruit present	***S. apetala***
–	Cup-shaped calyx, fruit absent	***S. × zhongcairongii***
8	Leaf blade apices rounded	***S. caseolaris***
–	Leaf blade apices apiculate, mucronate	***S. lanceolata***


## Supplementary Material

XML Treatment for S. × zhongcairongii
